# A Single Thermoresponsive Diblock Copolymer Can Form Spheres, Worms or Vesicles in Aqueous Solution

**DOI:** 10.1002/anie.201909124

**Published:** 2019-11-06

**Authors:** Liam P. D. Ratcliffe, Matthew J. Derry, Alessandro Ianiro, Remco Tuinier, Steven P. Armes

**Affiliations:** ^1^ Department of Chemistry, Dainton Building University of Sheffield Brook Hill South Yorkshire S3 7HF UK; ^2^ Laboratory of Physical Chemistry Department of Chemical Engineering & Institute for Complex Molecular Systems Eindhoven University of Technology P.O. Box 513, 5600 MB Eindhoven The Netherlands; ^3^ Present address: Unilever Research & Development Colworth Laboratory, Colworth House Sharnbrook Bedford MK44 1LQ UK

**Keywords:** block copolymers, nanoparticles, RAFT polymerization, self-assembly, thermoresponsivity

## Abstract

It is well‐known that the self‐assembly of AB diblock copolymers in solution can produce various morphologies depending on the relative volume fraction of each block. Recently, polymerization‐induced self‐assembly (PISA) has become widely recognized as a powerful platform technology for the rational design and efficient synthesis of a wide range of block copolymer nano‐objects. In this study, PISA is used to prepare a new thermoresponsive poly(*N*‐(2‐hydroxypropyl) methacrylamide)‐poly(2‐hydroxypropyl methacrylate) [PHPMAC‐PHPMA] diblock copolymer. Remarkably, TEM, rheology and SAXS studies indicate that a *single* copolymer composition can form well‐defined spheres (4 °C), worms (22 °C) or vesicles (50 °C) in aqueous solution. Given that the two monomer repeat units have almost identical chemical structures, this system is particularly well‐suited to theoretical analysis. Self‐consistent mean field theory suggests this rich self‐assembly behavior is the result of the greater degree of hydration of the PHPMA block at lower temperature, which is in agreement with variable temperature ^1^H NMR studies.

## Graphical abstract

**Every which way and loose**: A single amphiphilic thermoresponsive diblock copolymer can reversibly form spheres, worms or vesicles in aqueous solution. Self‐consistent mean field theory indicates that this rich self‐assembly behavior arises from the temperature‐dependent degree of hydration of the structure‐directing hydrophobic poly(2‐hydroxypropyl methacrylate) block.

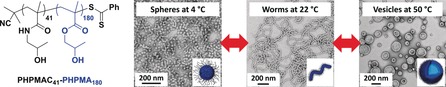

## Introduction

Block copolymer self‐assembly in solution has been known for more than fifty years.^1^ Many copolymer morphologies have been reported, including spheres, worms, rods, vesicles, lamellar platelets, disks, toroids, stomatocytes and framboidal vesicles.^2^ Potential applications include drug delivery, nanoencapsulation, membranes, biocompatible hydrogels, chemotaxis and diesel soot dispersion in engine oils.^3^ For the self‐assembly of amphiphilic AB diblock copolymer chains in aqueous solution, spheres,2c worms2f or vesicles^4^ are by far the most common copolymer morphologies. Such nano‐objects are now readily accessible via polymerization‐induced self‐assembly (PISA), which can be conducted in concentrated aqueous media.^5^ This is largely owing to the development of controlled radical polymerisation techniques such as reversible addition‐fragmentation chain transfer (RAFT) polymerization,^6^ which has enabled the convenient synthesis of a wide range of well‐defined functional block copolymers.^7^

In particular, many examples of thermoresponsive water‐soluble block copolymers have been reported in the literature.^4^, ^8^ Recently, PISA formulations have provided various examples of thermally‐induced worm‐to‐sphere, vesicle‐to‐sphere or vesicle‐to‐worm transformations.^9^ In the case of certain aqueous dispersions of thermoresponsive diblock copolymer nano‐objects, a worm‐to‐sphere or vesicle‐to‐sphere transition occurs on cooling. In contrast, for diblock copolymer nano‐objects dispersed in non‐aqueous media, a worm‐to‐sphere or vesicle‐to‐worm transition occurs on heating. Both phenomena can be explained in terms of surface plasticization of the insoluble structure‐directing block; this leads to a subtle change in the packing parameter^10^ that drives each morphological transition.^11^

**Figure 1:**
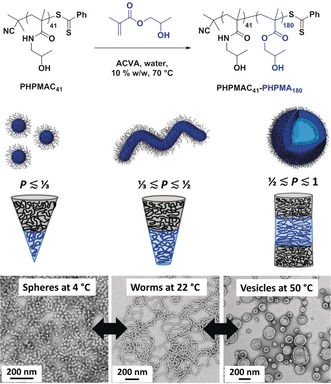
Synthesis of PHPMAC_41_‐PHPMA_180_ via RAFT aqueous dispersion polymerization of HPMA at 70 °C. Uniquely, this thermoresponsive diblock copolymer exhibits a double thermal transition enabling either spheres, worms or vesicles to be obtained at a fixed PHPMA DP, simply by varying the aqueous solution temperature from 4 °C to 50 °C (see TEM images and schematic cartoon).

Herein we report a new thermoresponsive AB diblock copolymer which exhibits remarkable self‐assembly behavior: a single copolymer composition that can form either spheres, worms or vesicles in aqueous solution depending solely on the temperature (see Figure 1). These two thermal transitions are again attributed to surface plasticization of the hydrophobic block, which becomes significantly more hydrated on lowering the aqueous solution temperature. The hydrophilic stabilizer block is a well‐known highly biocompatible polymer that has been extensively studied by others in the context of drug delivery applications: poly(N‐(2‐hydroxypropyl) methacrylamide) [PHPMAC].^12^ This water‐soluble polymer has been prepared by RAFT polymerization (see Figure S1a) with good control over its molecular weight distribution (MWD) being achieved.^13^ The hydrophobic structure‐directing block is poly(2‐hydroxypropyl methacrylate), which has been used for many PISA formulations based on RAFT aqueous dispersion polymerization.9b, 14 The remarkably subtle difference in chemical structure (see Figure 1) between these two types of monomer repeat units aids theoretical analysis of this system using self‐consistent mean field theory,^15^ because it ensures very similar segment volumes for these two components when using a lattice model.

## Results and Discussion

After conducting preliminary kinetic experiments to confirm a well‐controlled RAFT polymerization (see Figure S1), a PHPMAC_41_ macromolecular chain transfer agent (macro‐CTA) (*M*_n_=10,500, *M*_w_/*M*_n_=1.10) was prepared utilizing 2‐cyano‐2‐propyl benzodithioate (CPDB) for the RAFT solution polymerization of HPMAC in a 7:3 v/v 2‐propanol/water mixture at 25 % w/w. The reaction time was deliberately restricted to 4 h (41 % conversion by ^1^H NMR) to ensure high chain‐end fidelity. This water‐soluble precursor was then utilized to prepare a series of PHPMAC_41_‐PHPMA_y_ diblock copolymer nano‐objects at 10 % w/w solids via RAFT aqueous dispersion polymerization of HPMA at 70 °C, see Figure 1 and Figure S2.

Systematic variation of the target degree of polymerization (DP or *y*) for the hydrophobic PHPMA block from 140 to 220 led to the formation of either spheres, worms or vesicles at ambient temperature (22 °C) as judged by transmission electron microscopy (TEM) studies (see Figure S3). Similar observations have been reported for closely related aqueous PISA formulations in the literature.5b, 16 ^1^H NMR studies confirmed more than 99 % HPMA conversion within 3 h in each case while gel permeation chromatography (GPC; DMF eluent) indicated dispersities ranging from 1.13 to 1.20, suggesting well‐controlled RAFT polymerizations (see Figure S3).

On cooling from 70 °C to 22 °C, the physical appearance of some of the aqueous copolymer dispersions changed from a milky‐white free‐flowing fluid to either a gel or a less turbid fluid. Based on our prior experience,5b, 9a, 16 this suggested the likelihood of at least one thermally‐induced morphological transition. In particular, the PHPMAC_41_‐PHPMA_180_ copolymer dispersion exhibited three distinct physical states over a convenient temperature range: a weakly turbid fluid was obtained at 4 °C, a soft free‐standing gel was formed at 22 °C and a milky‐white free‐flowing dispersion was observed at 50 °C. Accordingly, the as‐synthesized 10 % w/w PHPMAC_41_‐PHPMA_180_ aqueous dispersion was diluted to 0.20 % w/w using deionized water, with each dilution being conducted at either 4 °C (with the aid of a refrigerator), 22 °C (ambient temperature) or 50 °C (with the aid of an oven), after allowing 24 h for equilibration. These dilute copolymer dispersions were analyzed by TEM (see Figure 1). Remarkably, this *single* PHPMAC_41_‐PHPMA_180_ diblock copolymer can form either spheres, worms or vesicles simply by varying the aqueous solution temperature: this involves crossing both the vesicle/worm and worm/sphere phase boundaries within a relatively narrow temperature range. Similar behavior has been recently reported by Delaittre and co‐workers^17^ for poly(2‐ethyl‐2‐oxazoline)‐PHPMA diblock copolymer nanoparticles. However, in this prior study morphological transitions were only shown to be reversible at a relatively high copolymer concentration (20 % w/w): the spheres formed at low temperature became kinetically‐trapped for thermal cycles performed in dilute solution (e.g. 0.1 % w/w copolymer). Moreover, the sole characterization data provided were TEM images obtained for dried diluted dispersions. In the current study, preliminary experiments indicated significant kinetic differences for the interconversion between spheres, worms and vesicles, even for 10 % w/w aqueous copolymer dispersions. More specifically, the worm‐to‐sphere transition occurred relatively quickly (within 45 min, according to rheological studies), whereas the complementary sphere‐to‐worm transition typically required rather longer time scales (hours). This is not particularly surprising given the former transition involves a dissociative mechanism (most likely via worm budding), whereas the latter requires a cooperative associative mechanism (i.e. multiple sphere‐sphere fusion events).9c Similar temporal differences were also observed for the vesicle‐to‐worm and worm‐to‐vesicle transitions (data not shown).

**Figure 2:**
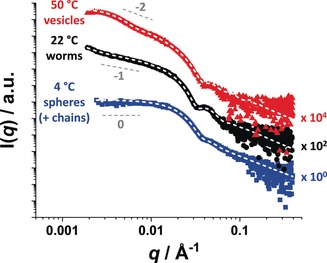
Small‐angle X‐ray scattering (SAXS) patterns recorded for 1.0 % w/w aqueous dispersions of PHPMAC_41_‐PHPMA_180_ diblock copolymer at 4 °C (blue squares), 22 °C (black circles) and 50 °C (red triangles), with an equilibration time of 24 h being allowed at each temperature. The white dashed lines shown represent data fits using (i) a linear combination of a spherical micelle model^19^ and a model for dissolved copolymer chains^23^ for the SAXS pattern recorded at 4 °C, (ii) a worm‐like micelle model^19^ for that recorded at 22 °C and (iii) a vesicle model^20^ for that recorded at 50 °C. [N.B. For clarity, the black and red curves are offset by arbitrary factors of 10^2^ and 10^4^, respectively].

Small‐angle X‐ray scattering (SAXS) is widely recognized as a powerful characterization technique for block copolymer nano‐objects.9a, 9g, 18 Unlike DLS, SAXS experiments can be performed on 10 % w/w copolymer dispersions and established models exist for the detailed analysis of spheres,^19^ worms^19^ and vesicles.^20^ Moreover, X‐ray scattering is averaged over millions of nano‐objects so the resulting structural information is far more statistically robust than that obtained from TEM image analysis. In view of these advantages, we performed a series of SAXS experiments. Initially, the following sample preparation protocol was adopted: 10 % w/w copolymer dispersions were equilibrated at the desired temperature for 24 h and then diluted ten‐fold with deionized water equilibrated at the same temperature. The resulting dilute dispersions were then immediately transferred to the pre‐equilibrated temperature‐controlled Linkam capillary cell and data were collected over 30 min. This series of experiments yielded three characteristic SAXS patterns, see Figure 2. There is no structure factor at a copolymer concentration of 1.0 % w/w, which simplifies the data analysis. The low *q* gradients for the patterns collected at 4 °C, 22 °C and 50 °C are consistent with the presence of spheres, worms and vesicles, respectively.^21^ Closer inspection suggests the presence of a significant proportion of dissolved copolymer chains (≈30 vol%) in addition to spheres at 4 °C. Similar findings have been reported for PHPMA‐based diblock copolymers at 3–5 °C by Kocik and co‐workers.^22^ Data fits to these SAXS patterns provided the mean sphere radius, worm cross‐sectional radius, overall vesicle radius and vesicle membrane thickness (see Table S1). Importantly, these SAXS data fits indicated that the volume fraction of solvent within the weakly hydrophobic PHPMA block increases on cooling from 50 °C to 4 °C.

Although fitting the SAXS data recorded at 22 °C provides a mean worm length, it is emphasized that our laboratory SAXS instrument does not provide access to sufficiently low *q* to enable accurate determination of this parameter. In addition, SAXS studies were conducted on 10 % w/w aqueous copolymer dispersions. Prominent structure factors were observed at this higher concentration for each morphology (data not shown), which complicates detailed analysis. Nevertheless, thermal cycling experiments (e.g. 22 °C to 4 °C to 22 °C and 22 °C to 50 °C to 22 °C) confirmed the thermoreversible nature of these morphological transitions because the initial and final SAXS patterns were remarkably similar at 10 % w/w (see Figure S4).

During PISA syntheses, spheres can be efficiently converted into worms within tens of minutes at elevated temperature.^16^, ^24^ However, this is because monomer swelling confers relatively high copolymer chain mobility under such conditions. In contrast, the morphological transitions observed in the present study occur in the absence of any unreacted monomer and are instead facilitated by the variable degree of hydration of the weakly hydrophobic PHPMA block. This interpretation is consistent with the relatively high volume fraction of water associated with this block indicated by the *x*_sol_ values obtained from SAXS data fits. In principle, variable temperature ^1^H NMR studies can provide further evidence for the hydrated nature of the structure‐directing PHPMA chains.

Accordingly, a 2.0 % w/w aqueous dispersion of PHPMAC_41_‐PHPMA_180_ diblock copolymer was freeze‐dried overnight and then redispersed in cold D_2_O (initially at 4 °C, following the protocol reported by Kocik et al.^22^) before warming up to ambient temperature. This dispersion was then used for ^1^H NMR spectroscopy studies at 50 °C, 22 °C and 4 °C (with 24 h being allowed at each temperature to achieve the preferred equilibrium morphology). Unfortunately, the very similar chemical structures of the PHPMAC and PHPMA blocks means that there is just one unique signal for the latter block. This corresponds to the two oxymethylene protons attached to the methacrylic ester at ≈4.1 ppm, which can be distinguished (but not fully resolved) from the two azamethylene protons of the PHPMAC stabilizer block at ≈3.9 ppm (see Figure S5). The relatively weak nature of the former signal indicates that the hydrophobic PHPMA block is only partially solvated at either 22 °C or 50 °C. However, this feature becomes more discernible at 4 °C, which is consistent with the variable temperature ^1^H NMR studies reported by Blanazs et al. for a closely‐related PHPMA‐based diblock copolymer.9a This greater degree of hydration at lower temperature is consistent with the observed change in morphology for the thermoresponsive PHPMAC_41_‐PHPMA_180_ diblock copolymer indicated in Figures 1 and 2 and has been rationalized in terms of a surface plasticization effect.5b

**Figure 3:**
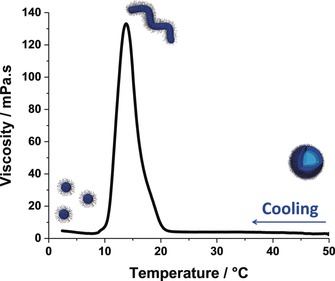
Temperature dependence of the viscosity obtained for a 10 % w/w aqueous dispersion of the PHPMAC_41_‐PHPMA_180_ diblock copolymer nano‐objects. The rheometer was pre‐heated to 50 °C and loaded with low‐viscosity PHPMAC_41_‐PHPMA_180_ vesicles. This dispersion was then cooled at a rate of 0.5 °C h^−1^. The local maximum in viscosity observed at around 14 °C indicates the formation of weakly interacting worms, while low‐viscosity spheres are obtained below 10 °C.

An as‐synthesized 10 % w/w aqueous dispersion of the PHPMAC_41_‐PHPMA_180_ diblock copolymer vesicles was prepared at 50 °C prior to being subjected to temperature‐dependent rheological studies. The rheometer was preheated to 50 °C and loaded with this low‐viscosity dispersion, which was then slowly cooled at a rate of 0.5 °C h^−1^ (see Figure 3). A local maximum in viscosity was observed at around 14 °C, which indicated the formation of weakly interacting worms. Cooling further to 2 °C led to the formation of low‐viscosity, non‐interacting spheres.

However, heating this cold dispersion of spheres revealed significant hysteresis (data not shown). This is because the dissociation of vesicles to form first worms and then spheres occurs on a relatively short time scale, but the reverse pathway is highly cooperative (e.g. worms are formed via the stochastic 1D fusion of multiple spheres^25^) and hence subject to relatively slow kinetics. Equilibrium copolymer morphologies can be eventually achieved on heating, but this requires relatively long time scales (typically many hours to days, depending on the copolymer concentration). Based on recent work by Warren and co‐workers,^26^ we anticipate that the viscosity maximum observed in Figure 3 should be tunable by systematically varying the mean DP of the PHPMA block. Indeed, this hypothesis is supported by tube inversion studies of a series of five PHPMAC_41_‐PHPMA_*x*_ diblock copolymers where *x* varies from 140 to 220 (see Figure S6). Theoretical calculations for the effect of varying this parameter from 50 to 300 are also shown in the Supporting Information (see Figure S7). However, further rheological experiments to verify these predictions are beyond the scope of the current study.

In order to understand the underlying mechanism that drives these morphological transitions, we performed calculations of the equilibrium properties of these self‐assembled structures using numerical lattice computations based on the self‐consistent field (SCF) theory developed by Scheutjens and Fleer.^15^ This approach is based on Flory–Huggins lattice theory^27^ and small‐system thermodynamics.^28^ It utilizes a theoretical mean field model, where the block copolymers and the solvent molecules are distributed over a three‐dimensional lattice, while accounting for concentration gradients in one direction. Given a specific copolymer composition, the change in the configurational entropy upon mixing polymer and solvent is calculated via a step‐weighted random walk while the enthalpy of mixing is modeled via a set of pair interaction parameters, also known as *χ* parameters.15b, 27 This approach enables various thermodynamic properties to be calculated, including the preferred copolymer morphology, the mean aggregation number for the self‐assembled copolymer chains (*g*), and the surface area occupied by each copolymer chain at the core/corona interface (*s*), as well as concentration profiles for all components inside and outside the self‐assembled structure.

For the present work, three *χ* parameters must be considered: *χ*_HPMA‐W_, *χ*_HPMAC‐HPMA_ and *χ*_HPMAC‐W_. Both *χ*_HPMAC‐HPMA_ and *χ*_HPMAC‐W_ are held constant: the former parameter is taken to be unity to ensure inter‐block segregation and, according to the literature, the latter parameter has a numerical value of 0.48.^29^ This condition is necessary because, if the blocks were not segregated, SCF modeling predicts macroscopic phase separation rather than colloidally‐stable diblock copolymer nano‐objects. *χ*_HPMA‐W_ has been estimated to be 0.83 at room temperature utilizing the method proposed by Lindvig et al.^30^ This approach uses the Hansen solubility parameter and is consistent with experimental values obtained for similar molecules.^31^ To simulate temperature variation, the *χ* parameter of the thermoresponsive block (*χ*_HPMA‐W_) is varied between 0.50 and 1.50 with a step size of 0.02. This interval is considered most relevant for morphology transitions because for *χ*_HPMA‐W_<0.50 the copolymer is expected to be fully soluble, while the copolymer morphology is expected to be kinetically frozen for such a relatively long hydrophobic PHPMA block if *χ*_HPMA‐W_>1.50. For such theoretical calculations, the diblock copolymer chains are assumed to be perfectly uniform in chain length. Recently, Ianiro et al. reported that a reasonably narrow chain length distribution has a negligible effect on their self‐assembly in solution.^32^ The equilibrium morphology is determined at each step by performing the calculations for lattices with different geometries (spherical, cylindrical and flat). The preferred copolymer morphology corresponds to the geometry with the lowest critical micellization concentration (CMC), since the Gibbs free energy of micellization^33^ can be approximately expressed as:

(1) $$\Delta G{}^{\mathrm{mic}}\approx R\;T\;\ln\left(\mathrm{CMC}\right)$$

**Figure 4:**
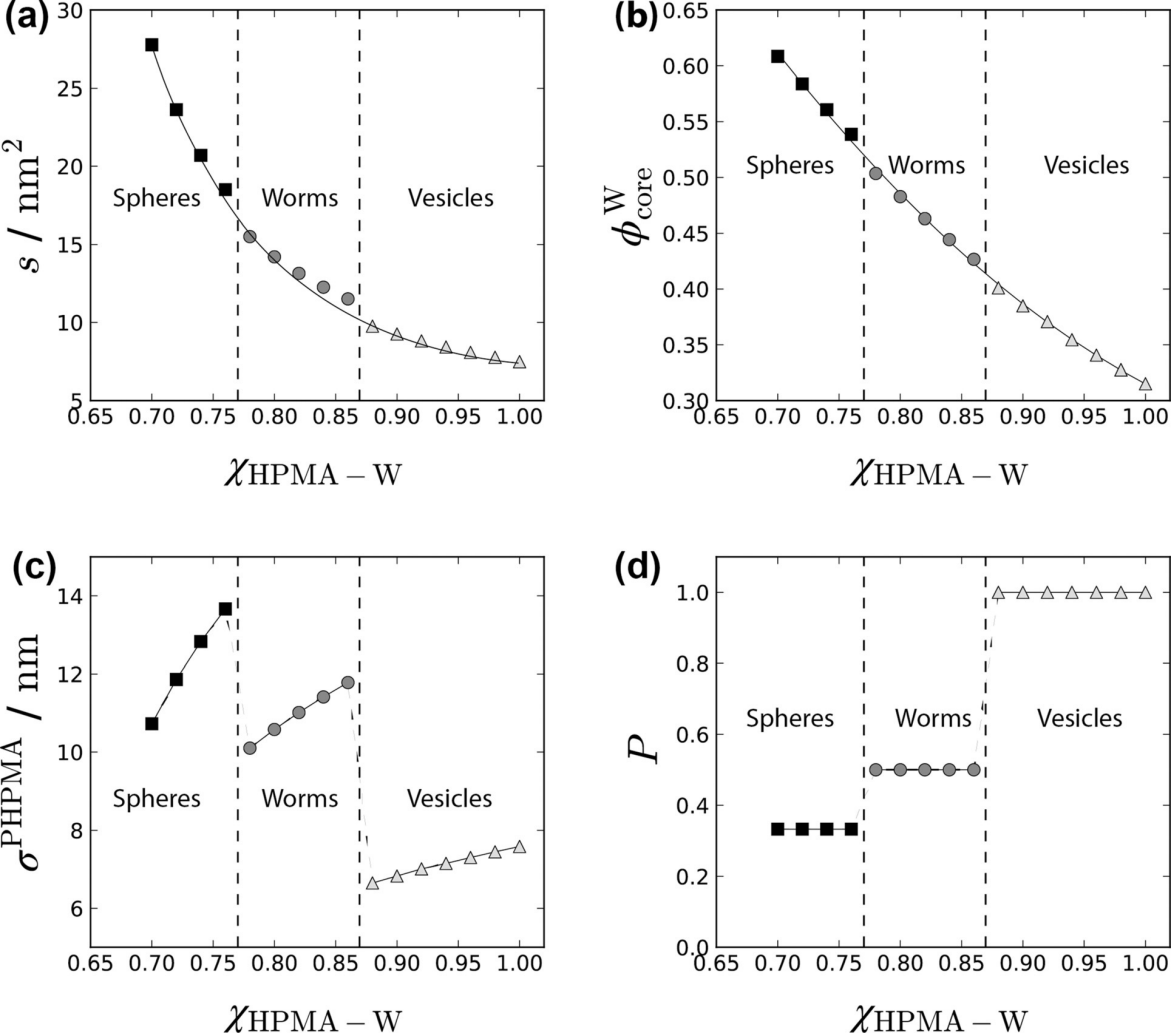
Variation of a) the interfacial surface area per copolymer chain (*s)*, b) the average volume fraction of water associated with the hydrophobic core‐forming PHPMA block $\left(\phi_{\mathrm{core}}^{W}\right)$ , c) the average end‐to‐end distance of the PHPMA block (*σ* ^PHPMA^) and d) the molecular packing parameter *P* as a function of *χ*_HPMA‐W_, as calculated for PHPMAC_41_‐PHPMA_180_. Dashed lines mark the sphere/worm and worm/vesicle boundaries.

According to our SCF calculations (see Figure 4), the PHPMAC_41_‐PHPMA_180_ copolymer preferentially assumes a spherical morphology if 0.70<*χ*_HPMA‐W_<0.78, while cylindrical (or worm‐like) micelles are the thermodynamically preferred state for the 0.78<*χ*_HPMA‐W_<0.88 interval. For *χ*_HPMA‐W_>0.88, SCF theory predicts vesicle formation.

Although more precise knowledge of the interaction parameters and their temperature dependence is required for a *quantitative* comparison between our model and the experimental data, these results *qualitatively* describe the morphological transitions that are observed experimentally.

The interfacial tension (*γ*) at the core‐corona interface of the diblock copolymer micelles may be estimated from the interaction parameter between the solvent and the solvophobic block *χ*_HPMA‐W_ . It follows from theory^34^ that *γ*≈(*χ*_HPMA‐W_)^1/2^. In turn, the solvophobic block–water interaction *χ*_HPMA‐W_ increases with temperature, which is consistent with the ^1^H NMR spectra shown in Figure S5. Hence, at low temperatures (4 °C), *γ* is small and the copolymer morphologies are characterized by a high equilibrium value for the interfacial surface area (*s*) [see Eq. (S2)–(S4) in the Supporting Information]. This results in much lower steric repulsion between the chains in the core‐forming block, which reduces the degree of chain stretching and hence favors a spherical morphology. At higher temperature, the increase in *γ* necessitates a reduction in *s* (see Figure 4 a). This results in expulsion of water molecules from the core (see Figure 4 b) and in a gradual increase in the end‐to‐end distance (*σ* ^PHPMA^) of the core blocks (see Figure 4 c). On further increasing *σ* ^PHPMA^, chain stretching becomes energetically too unfavorable to maintain the spherical morphology, resulting in a transition to form first worms at 22 °C and then vesicles at 50 °C. These morphology transitions enable a reduction of *σ* ^PHPMA^ (see Figure 4 c) and hence a reduction in the overall free energy (Δ*G*) of the system. The copolymer packing parameter (*P*) calculated according to Equation S8 (see Supporting Information) is plotted in Figure 4 d.^35^ The fractional values obtained for spheres, worms and vesicles are consistent with the literature.^35^

## Conclusion

In summary, we report a new thermoresponsive amphiphilic diblock copolymer that can form spheres, worms or vesicles in aqueous solution simply by varying the solution temperature. This unprecedented self‐assembly behavior is driven by the variable degree of hydration of the core‐forming poly(2‐hydroxypropyl methacrylate) block, which enables two phase boundaries to be crossed within a relatively narrow temperature range. Theoretical analysis of this new diblock copolymer system using self‐consistent mean field theory supports our experimental observations. Finally, we envisage that the worm‐to‐vesicle thermal transition reported herein should provide new opportunities for the convenient loading of nanoparticles, proteins or enzymes within vesicles. Moreover, the vesicle‐to‐worm transition that is observed on cooling could provide a suitable (and tunable) release mechanism for such payloads.

## Conflict of interest

The authors declare no conflict of interest.

## Supporting information

As a service to our authors and readers, this journal provides supporting information supplied by the authors. Such materials are peer reviewed and may be re‐organized for online delivery, but are not copy‐edited or typeset. Technical support issues arising from supporting information (other than missing files) should be addressed to the authors.

Supplementary
