# Supplementary material for: A Single Thermoresponsive Diblock Copolymer Can Form Spheres, Worms or Vesicles in Aqueous Solution
Source: Angew Chem Int Ed Engl. 2019 Nov 6;58(52):18964–70. doi: 10.1002/anie.201909124 (PMC6973111; doi:10.1002/anie.201909124)
Supplement: Supplementary file 1 — Supplementary [file ANIE-58-18964-s001.pdf]

Angewandte Chemie
Eine Zeitschrift der Gesellschaft Deutscher Chemiker

Supporting Information

A Single Thermoresponsive Diblock Copolymer Can Form Spheres, Worms or Vesicles in Aqueous Solution

Liam P. D. Ratcliffe[+]*, Matthew J. Derry*[+]*, Alessandro Ianiro*[+]*, Remco Tuinier,* and Steven P. Armes**

anie_201909124_sm_miscellaneous_information.pdf

Supporting Information for:

A Single Thermoresponsive Diblock Copolymer Can Form Spheres, Worms or Vesicles in Aqueous Solution

Liam P. D. Ratcliffe,[$] Matthew J. Derry,[$] Alessandro Ianiro,[$] Remco Tuinier,* and Steven P. Armes*

Experimental Section and Methods

Materials

2-Hydroxypropyl methacrylate (HPMA, 97 %) and 4,4'-azobis(4-cyanopentanoic acid) (ACVA; V-501; 99 %) were purchased from Alfa Aesar (Heysham, UK) and used as received. *N*-(2-Hydroxypropyl) methacrylamide (HPMAC) was synthesized in-house as described by Jay *et al*.[1] 2-Cyano-2-propyl benzodithioate (CPDB) was purchased from Strem Chemicals (Cambridge, UK). 1-Amino-2-propanol (93 %), methacryloyl chloride (≥ 97 %) and deuterium oxide (D_2O) were purchased from Sigma-Aldrich (Dorset, UK). Deuterated methanol (CD_3OD) was purchased from Goss Scientific (Nantwich, UK). Sodium hydroxide pellets (NaOH) were purchased from VWR (Lutterworth, UK). Deionized water was used for all RAFT aqueous dispersion polymerization syntheses, as well as for characterization of aqueous copolymer dispersions. All other solvents were HPLC-grade, purchased from Fisher Scientific (Loughborough, UK) and used as received, unless stated otherwise.

Synthesis and purification of PHPMAC monomer

The HPMAC monomer was synthesized and purified according to the literature.[1]

Synthesis and purification of PHPMAC$_{41}$ macromolecular chain transfer agent (macro-CTA)

A typical protocol for the synthesis of PHPMAC$_{41}$ macro-CTA is as follows. HPMAC monomer (0.11 mol, 15.7498 g) and ACVA initiator (0.28 mmol, 0.0771 g) were dissolved in water (0.803 mol, 14.46 g) in a round-bottomed flask and the pH was adjusted with HCl to 4.0. CPDB RAFT agent (1.10 mmol, 0.2434 g; CTA/ACVA molar ratio = 4.0; target degree of polymerization, DP = 100) dissolved in propan-2-ol (33.75 g, 0.562 mol) was added to this reaction flask and the resulting pink solution was purged with N_2 in ice for 30 min, before the sealed flask was

immersed into an oil bath set at 70 °C. After 4 h (41 % conversion as judged by ^{1}H NMR) the polymerization was quenched by immersing the flask in an ice bath and exposing the reaction solution to air. The crude polymer solution was then precipitated into a ten-fold excess of diethyl ether three times before being dissolved in water and lyophilized overnight. ^{1}H NMR analysis indicated a mean DP of 41 for this PGMA macro-CTA. Taking into account the target DP of 100 and the monomer conversion of 41 %, this suggests a CTA efficiency of ~100 %. DMF GPC analysis (refractive index detector; relative to a series of poly(methyl methacrylate) calibration standards) indicated M_n and M_w/M_n values of 14,300 g mol^{-1} and 1.14, respectively.

Synthesis of PHPMAC$_{41}$-PHPMA$_{180}$ diblock copolymer via RAFT aqueous dispersion polymerization of HPMA.

A typical protocol for the synthesis of PHPMAC$_{41}$-PHPMA$_{180}$ diblock copolymer is as follows: PHPMAC$_{41}$ macro-CTA (0.300 g, 0.049 mmol), followed by HPMA monomer (1.278 g, 8.86 mmol; target DP = 180), and water (14.20 g, to produce a 10 % w/w solution) were added to a glass vial. ACVA was then added (3.50 mg, 0.012 mmol, macro-CTA/ACVA molar ratio = 4.0) and the solution was purged with N_2 for 30 minutes. The flask was sealed, immersed in an oil bath set at 70 °C and stirred for 3 h. The polymerizing solution was quenched by exposure to air and cooling the flask to 20 °C. ^{1}H NMR analysis indicated >99 % HPMA conversion.

^{1}H NMR spectroscopy

For monomer conversion determination, (co)polymers were dissolved in deuterated methanol (CD_3OD) and ^{1}H NMR spectra were recorded using a 400 MHz Bruker Avance spectrometer (32 scans averaged per spectrum). For variable-temperature ^{1}H NMR spectroscopy studies, the freeze-dried PHPMAC$_{41}$-PHPMA$_{180}$ diblock copolymer was dispersed in D2O at 5 % w/w and equilibrated at the desired temperature for 24 h before spectra were recorded at 4, 22 or 50 °C using a Bruker AVIII 400 MHz spectrometer (32 scans averaged per spectrum).

Gel permeation chromatography (GPC)

Molecular weights and dispersities were determined using a DMF GPC instrument operating at 60 °C. The set-up comprised two Polymer Laboratories PL gel 5 µm Mixed C columns and one PL polar gel 5 µm guard column connected in series to an Agilent Technologies 1260 Infinity multi-detector suite and an Agilent Technologies 1260 Infinity pump injection module. The GPC eluent was HPLC-grade DMF containing 10 mM LiBr and was filtered prior to use. The flow rate was 1.0 ml min^{-1} and DMSO was used as the flow-rate marker. Calibration was conducted using a series of ten near-monodisperse poly(methyl methacrylate) standards (M_n = 625 – 618,000 g mol^{-1}). Chromatograms were analyzed using Agilent Technologies GPC/SEC software version 1.2.

Transmission electron microscopy (TEM)

0.20 % w/w copolymer dispersions were prepared at 4, 22 or 50 °C. Samples at 4 °C were prepared in a fridge set to this temperature and those at 22 °C or 50 °C in an oven. Copper/palladium TEM grids (Agar Scientific, UK) were surface-coated in-house to produce a thin film of amorphous carbon, then plasma glow-discharged for 30 s to create a hydrophilic surface. Droplets of freshly-prepared aqueous copolymer dispersions at the relevant temperature (9 µL; 0.20 % w/w) were placed on a hydrophilic grid for 1 min and then blotted with filter paper to remove excess solution. The deposited nanoparticles were then negatively stained with an aqueous solution of uranyl formate (9 µL; 0.75 % w/w) for a further 20 s, then carefully blotted to remove excess stain and dried with a vacuum hose. TEM grids were imaged using a FEI Tecnai Spirit TEM instrument equipped with a Gatan 1kMS600CW CCD camera operating at 120 kV.

Dynamic light scattering (DLS)

Analyses were conducted at 4, 22, 50 or 70 °C using a Malvern Instruments Zetasizer Nano series instrument equipped with a 4 mW He–Ne laser (λ = 633 nm) and an avalanche photodiode detector. Scattered light was detected at 173°. Copolymer dispersions were diluted to 0.20 % w/w using an aqueous solution of 1 mM KCl (pH 8), and the solution pH was adjusted using aqueous KOH as required. Intensity-average hydrodynamic diameters were calculated via the Stokes–Einstein equation. Zeta potentials were calculated from the Henry equation using the Smoluchowski approximation.

Small-angle X-ray scattering (SAXS)

SAXS data were collected using a laboratory SAXS instrument (Xeuss 2.0, Xenocs, France) equipped with a liquid gallium MetalJet X-ray source (Excillum, Sweden, wavelength λ = 0.134 nm), with motorized scatterless slits for beam collimation and a Dectris Pilatus 1M pixel detector (sample-to-detector distance 1.889 m or 6.335 m). Where SAXS data were collected at both sample-to-detector distances, data were combined to produce scattering patterns over a q range of 0.003 $Å^{-1} < q < 0.4$ $Å^{-1}$, where $q = 4\pi \sin \theta/\lambda$ is the scattering vector and θ is one-half of the scattering angle). Samples were equilibrated at the required temperature for 24 h before being transferred to a glass capillary of 2 mm diameter. The temperature was then controlled using a heating/cooling capillary holding stage (Linkam Scientific Instruments Ltd., Tadworth, UK). Data were collected for 10 min and averaged over three measurements before being reduced using the Foxtrot software package developed by SOLEIL and further analyzed using Irena SAS macros[2] for Igor Pro.

Rheology

Measurements were performed using an Anton Paar MCR502 rheometer equipped with a Peltier temperature controller, cone-and-plate geometry (a truncated 50 mm 2° stainless steel cone) and TruGap functionality for online monitoring of the geometry gap. A 10 % w/w aqueous dispersion of the $PHPMAC_{41}$-$PHPMA_{180}$ diblock copolymer nano-objects was equilibrated at 50 °C before being transferred to the rheometer, with the viscosity measured

during a 50-2 °C thermal sweep at a cooling rate of 0.5 °C h^{-1}. The continuous shear rate was fixed at 10 s^{-1}.

Self-consistent field (SCF) calculations

SCF computations have been performed using the SFbox,[3] provided by Prof. F.A.M. Leermakers, Wageningen University, the Netherlands. In the SCF procedure the i components of a mixture, each comprising N_i molecules, are distributed over a lattice. An iterative procedure is used to compute the spatial distribution of the components that minimizes the free energy of the lattice F. The value of F is used together with the chemical potentials μ_i of the components to compute the grand potential function $\Omega = F - \sum_i \mu_i N_i$ The settings of the boundary conditions, the lattice and interaction parameters derivation are as follows:

Lattice and boundary conditions

SCF computations were performed on lattices containing $L = 100$ lattice layers in the gradient direction and spherical, cylindrical and flat geometries were used to study spherical micelles, cylindrical micelles and vesicles, respectively. A lattice coordination number, z, of 3 was used in each case. For the flat geometry, two mirrors are placed at the first and last lattice layer. For the spherical and cylindrical geometries, a mirror was placed at the last lattice layer (the first one, corresponding to the centre of the lattice, was not used for computing the concentration gradients and its composition was assumed to be equal to that of the second lattice layer). In the lattice, each solvent molecule (W) and the monomer repeat units of the PHPMAC and PHPMA blocks are modeled such that they occupy a single lattice site. The diblock copolymer chains are assumed to be perfectly uniform and the system is assumed to be neutral. Each lattice site was assumed to correspond to the size of a single PHPMA monomer repeat unit, l. A numerical value for l was estimated to be 0.6 nm by applying the relation:

$$\boldsymbol{l} = \left(\frac{M_{\mathrm{HPMA}}}{\rho N_{\mathrm{AV}}}\right)^{\frac{1}{3}} \qquad (\boldsymbol{S}1)$$

where M_{HPMA} is the molar mass of the HPMA monomer (144.17 g·mol^{-1}), ρ is the density of the PHPMA block ($\rho = 1.21\ \mathrm{kg \cdot m^{-3}} = 1.21\,.\,10^{-21}\mathrm{g \cdot nm^{-3}}$) and N_{AV} is Avogadro's number.

The solvent volume fraction in the core, $\phi^{\mathrm{W}}_{\mathrm{core}}$, can be estimated from the solvent equilibrium concentration profile at the center of the insoluble PHPMA core. From the value of $\phi^{\mathrm{W}}_{\mathrm{core}}$ and the aggregation number ($N^{\mathrm{SCF}}_{\mathrm{i}}$, with i indicating the different morphologies) provided by the SCF computations, the surface area (s) occupied by each polymer at the core-corona interface can be calculated. On the basis of the different lattice symmetries, it can be shown that, for spheres, *s* is given by:

$$\boldsymbol{s} = \left(\frac{36\pi n^2 l^6}{\left(1-\phi^{\mathrm{W}}_{\mathrm{core}}\right)^2 N^{\mathrm{SCF}}_{\mathrm{S}}}\right)^{\frac{1}{3}} \qquad \boldsymbol{S}2$$

Similarly, for cylinders, *s* is given by:

$$s = \left(\frac{4\pi n l^4}{(1-\phi_{\mathrm{core}}^{\mathrm{W}})N_{\mathrm{W}}^{\mathrm{SCF}}}\right)^{\frac{1}{2}} \qquad \mathbf{S}3$$

Finally, for a planar geometry (which corresponds to bilayers or vesicles), *s* is given by:

$$s = \frac{2l^2}{N_{\mathrm{V}}^{\mathrm{SCF}}} \qquad \mathbf{S}4$$

The term *n* is the mean degree of polymerization of the PHPMA block. Such expressions are derived from the ratio between total interfacial area of the assemblies and aggregation number. It is noted that the in the SCF computations the definition of $N_{\mathrm{i}}^{\mathrm{SCF}}$ is geometry-dependent: $N_{\mathrm{S}}^{\mathrm{SCF}}$ is the number of amphiphilic copolymer chains forming each micelle; $N_{\mathrm{W}}^{\mathrm{SCF}}$ is the number of molecules contained within a slice of the worm-like micelle with a thickness equal to the lattice constant l ; $N_{\mathrm{V}}^{\mathrm{SCF}}$ is the number of copolymer molecules per surface area. This definition differs from the one used in the modelling of the experimental SAXS data (Equations S21, S29, and S32).

The core-end distance of the PHPMA blocks, σ^{PHPMA}, could be in principle determined by the concentration profiles from the thickness of the PHPMA core. The existence of a core-corona interface, however, makes difficult to formally define the boundary of the core region. Therefore, σ^{PHPMA} have been derived using the geometric arguments described above. The resulting expressions are:

$$\sigma^{\mathrm{PHPMA}} = \frac{3l^3 n}{s(1-\varphi_{\mathrm{S}}^{\mathrm{core}})} \qquad \mathbf{S}5$$

for spheres,

$$\sigma^{\mathrm{PHPMA}} = \frac{2l^3 n}{s(1-\varphi_{\mathrm{S}}^{\mathrm{core}})} \qquad \mathbf{S}6$$

for cylinders, and

$$\sigma^{\mathrm{PHPMA}} = \frac{l^3 n}{s(1-\varphi_{\mathrm{S}}^{\mathrm{core}})} \qquad \mathbf{S}7$$

for a planar geometry (bilayers or vesicles).

The packing parameter *P* is defined as:

$$P = \frac{V}{\sigma s} \qquad \mathbf{S}8$$

where *V* is the volume of a solvophobic block. In 1976 Israelachvili and co-workers introduced the concept of the fractional packing parameter *P* to account for the self-assembly of small molecule surfactants.[4] Subsequently, this concept has been extended to include block copolymer self-assembly.[5] From SCF computations *P* can be estimated as:

$$\boldsymbol{P} = \frac{l^3 n}{s\sigma^{\text{PHPMA}}(1-\phi_{\text{core}}^{\text{W}})} \qquad \boldsymbol{S}9$$

where l^3 is assumed to be the volume of one PHPMA monomer. In Equation (9), $l^3 n/(1-\phi_{\text{core}}^{\text{W}})$ represents the volume of the partially hydrated PHPMA block. By substituting s and σ^{PHPMA} in Equation (9), the expected values of the packing parameter $P = {}^{1}/_{3}$ in the spherical morphology, $P = {}^{1}/_{2}$ in the cylindrical morphology and $P = 1$ in the planar morphology, are obtained.

Dependence of the χ parameter

Given its enthalpic nature, the χ parameter depends on the temperature, but the precise mathematical form of this dependence is related to the chemical composition of the interacting pair of monomer repeat units and does not follow a general rule. In absence of specific interactions, χ often decreases with increasing temperature according to the (empirical) relation:

$$\chi(\boldsymbol{T}) = \mathbf{A} + \frac{\mathbf{B}}{\boldsymbol{T}} \qquad \boldsymbol{S}10$$

where A and B are numerical constants and *T* is the absolute temperature.

When specific interactions such as hydrogen bonding are involved, the situation becomes far more complicated, and various types of temperature dependence can be observed. The number of hydrogen-bonded solvent molecules in the first and second solvation layer around the copolymer chains is generally reduced with increasing temperature; this results in a lower degree of solvation, as experimentally observed for many systems.[6] To model this effect, $\chi(T)$ can be considered to comprise two contributions: the first accounts for non-specific interactions (which are inversely proportional to temperature), while the second accounts for the hydrogen bonding (which is proportional to temperature). This situation is represented by Equation (9):

$$\chi(\boldsymbol{T}) = \boldsymbol{\alpha}\left(\frac{1}{T}\right) + \boldsymbol{\beta}(\boldsymbol{T}^{\mathbf{i}}) \qquad \boldsymbol{S}11$$

for $i > 0$. If the first term dominates, then $\chi(T)$ decreases with temperature. If the two contributions are balanced, then $\chi(T)$ may be only weakly dependent on temperature, or it may first increase then decrease, giving rise to the so-called closed-loop phase behavior.[7]

Effect of varying the PHPMA DP on the copolymer morphology transitions.

"To understand how the copolymer morphology transitions are affected by the chain length of the thermoresponsive structure-directing block, additional calculations were performed in which the PHPMA block DP was systematically varied from $n = 50$ to $n = 300$. As shown in

Figure S6 on page S13, the $\chi_{\mathrm{HPMA-W}}$ values (and hence the temperatures) corresponding to the chain-to-sphere, sphere-to-cylinder and cylinder-to-vesicle morphology transitions increase for shorter PHPMA blocks. Simultaneously, the stability windows for the different morphologies are also affected. For $n \lesssim 90$ the cylinder-to-vesicle transition becomes inaccessible within the investigated range of $\chi_{\mathrm{HPMA-W}}$ values. The same observation is made for the sphere-to-cylinder transition when $n \lesssim 55$. These results indicate that the thermoresponsive behavior of this diblock copolymer system can be tuned by varying the PHPMA DP. A sufficiently long PHPMA block provides convenient access to all three copolymer morphologies over a relatively narrow range of $\chi_{\mathrm{HPMA-W}}$ values and stabilizes the vesicle morphology. Conversely, only spheres are accessible for shorter PHPMA blocks."

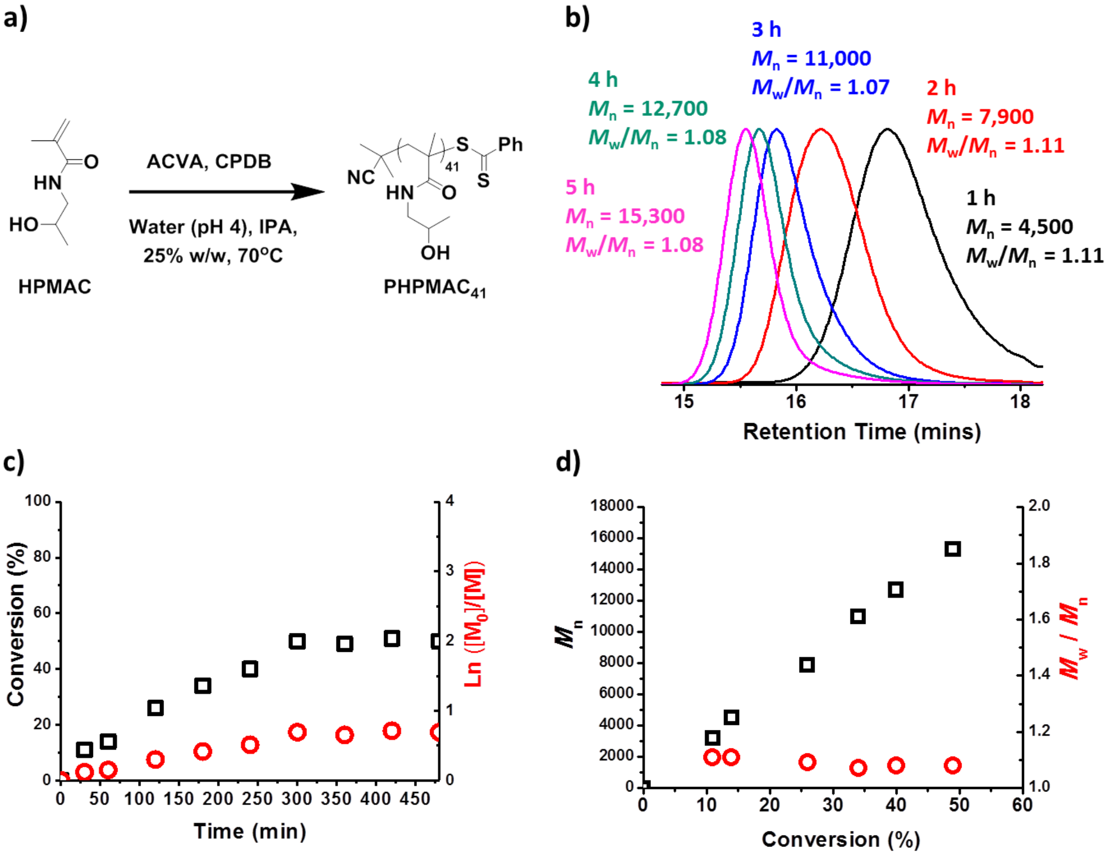

Figure S1. (a) Reaction scheme for the synthesis of a $PHPMAC_{41}$ macro-CTA at 70°C *via* RAFT solution polymerization of HPMAC in a 7:3 v/v IPA:water mixture at 25 % w/w solids, utilizing a CPDB/ACVA molar ratio of 4.0. Kinetic data obtained for this polymerization: (b) DMF GPC curves recorded after the stated reaction time. (c) Conversion *vs.* time curve (black squares) and corresponding semi-logarithmic plot (red circles). (d) Number-average molecular weight (black squares) *vs*. conversion and the corresponding dispersity *vs*. conversion plots. A linear evolution in molecular weight is observed and dispersities remain low ($M_w/M_n \leq 1.11$), suggesting a well-controlled RAFT polymerization.

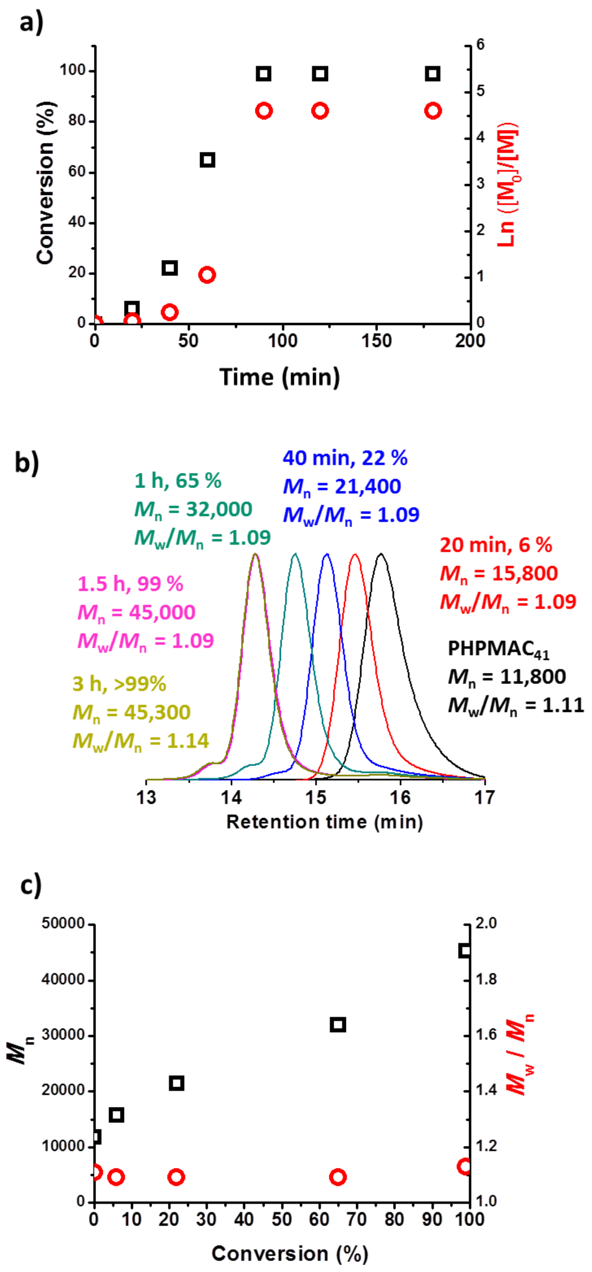

Figure S2. Kinetic data obtained for the synthesis of a $PHPMAC_{41}$-$PHPMA_{180}$ diblock copolymer *via* aqueous dispersion polymerization at 10 % w/w solids, 70°C and utilizing a CTA/initiator molar ratio of 4.0. (a) Conversion *vs.* time curve (black squares) and the corresponding semi-logarithmic plot (red circles). (b) DMF GPC curves recorded after the stated reaction time and corresponding monomer conversion, as calculated using ^{1}H NMR spectroscopy. (c) Number-average molecular weight (black squares) and copolymer dispersity *vs*. conversion plots determined by combining GPC and ^{1}H NMR data, respectively. A linear evolution of molecular weight is observed and M_w/M_n values always remain below 1.15, suggesting a well-controlled RAFT polymerization.

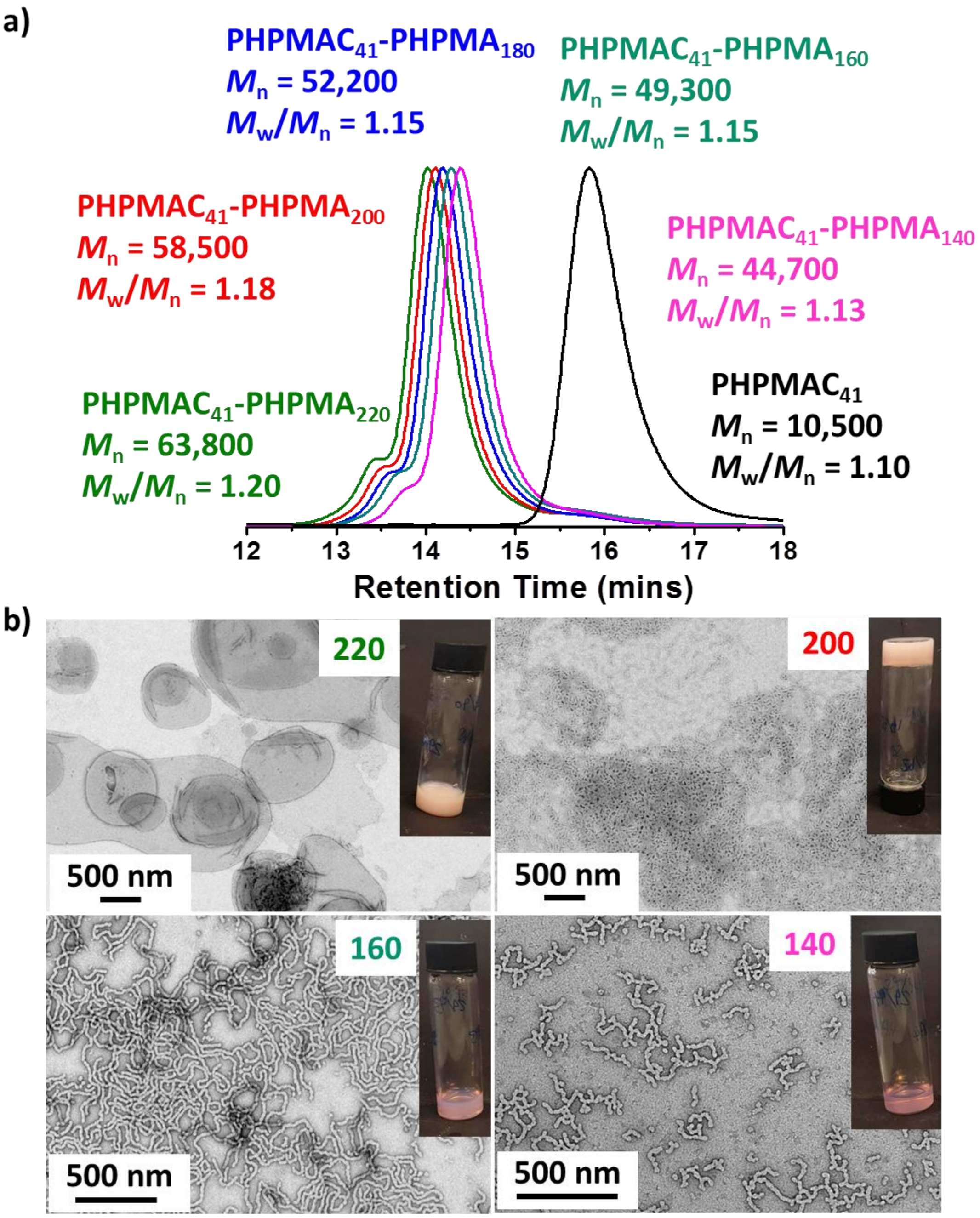

Figure S3. DMF GPC curves recorded for PHPMAC$_{41}$-PHPMA$_y$ diblock copolymers *via* RAFT aqueous dispersion polymerization of HPMA at 10 % w/w and 70 °C. Below are the physical appearances of these gels and TEM images made at 22 °C. [N.B. The high molecular weight shoulder observed in these GPC curves is attributed to a well-known dimethacrylate impurity that is present in HPMA monomer. This inevitably leads to light branching when targeting higher PHPMA DPs (as shown in Figure S3a above)].

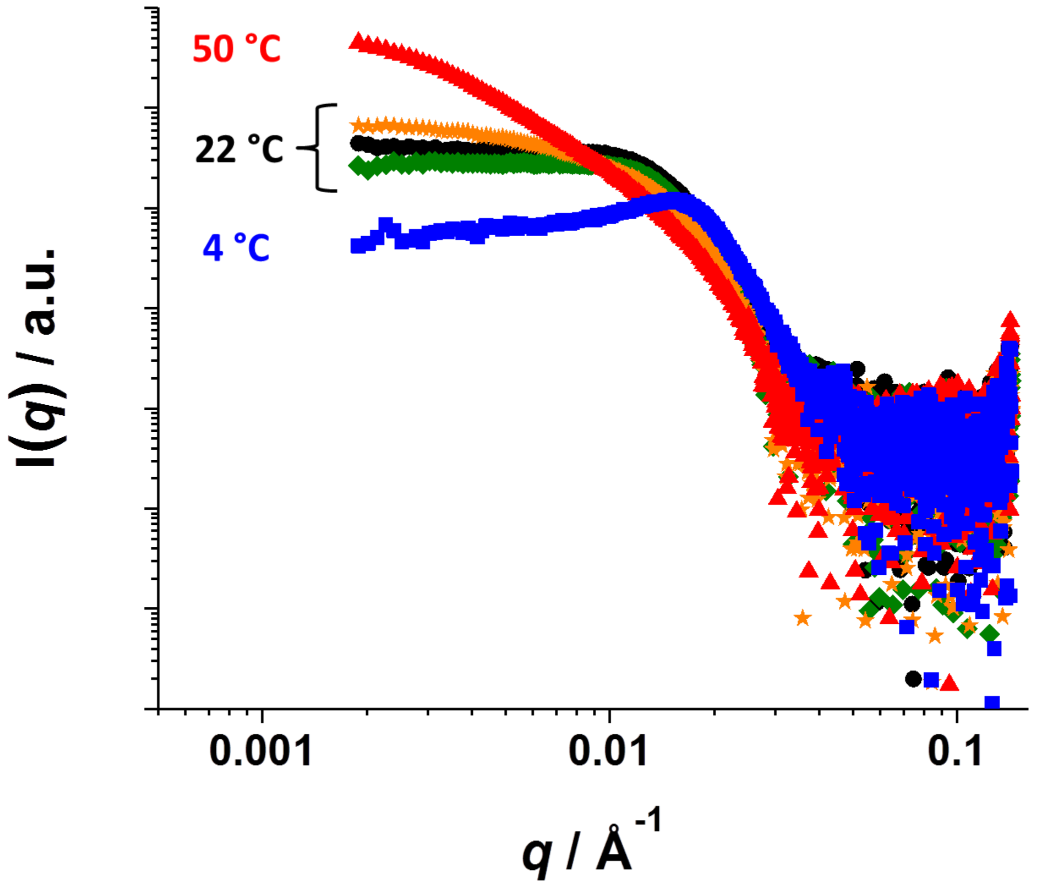

Figure S4. Small-angle X-ray scattering data collected for a 10% w/w aqueous dispersion of $PHPMAC_{41}$-$PHPMA_{180}$ diblock copolymer nanoparticles during a thermal cycle starting at 22 °C (black circles), before cooling to 4 °C (blue squares), then returning to 22 °C (green diamonds), then heating to 50 °C (red triangles) and finally on cooling to 22 °C (orange stars). In all cases, 24 h equilibration was allowed prior to data collection.

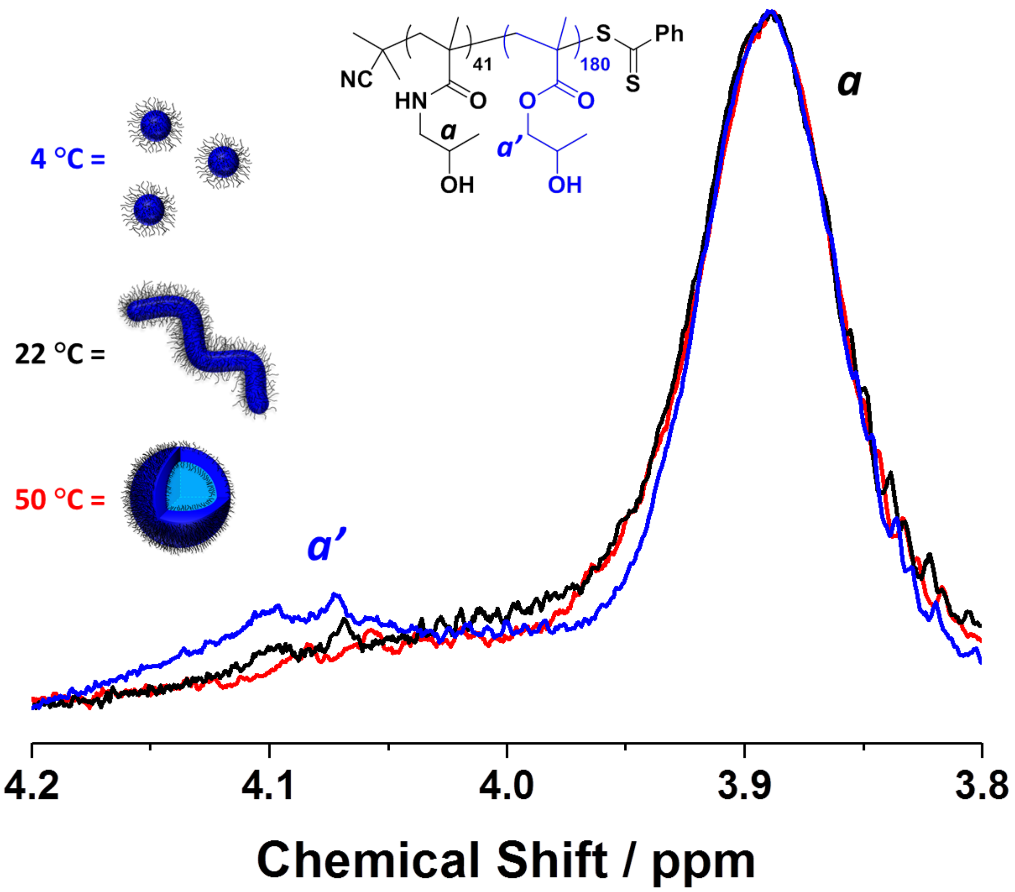

Figure S5. Assigned partial ^{1}H NMR spectra recorded for a 5.0% w/w dispersion of $PHPMAC_{41}$-$PHPMA_{180}$ diblock copolymer nanoparticles in D_2O at 50 °C (red data), 22 °C (black data) and 4 °C (blue data) after equilibration for 24 h at the desired temperature.

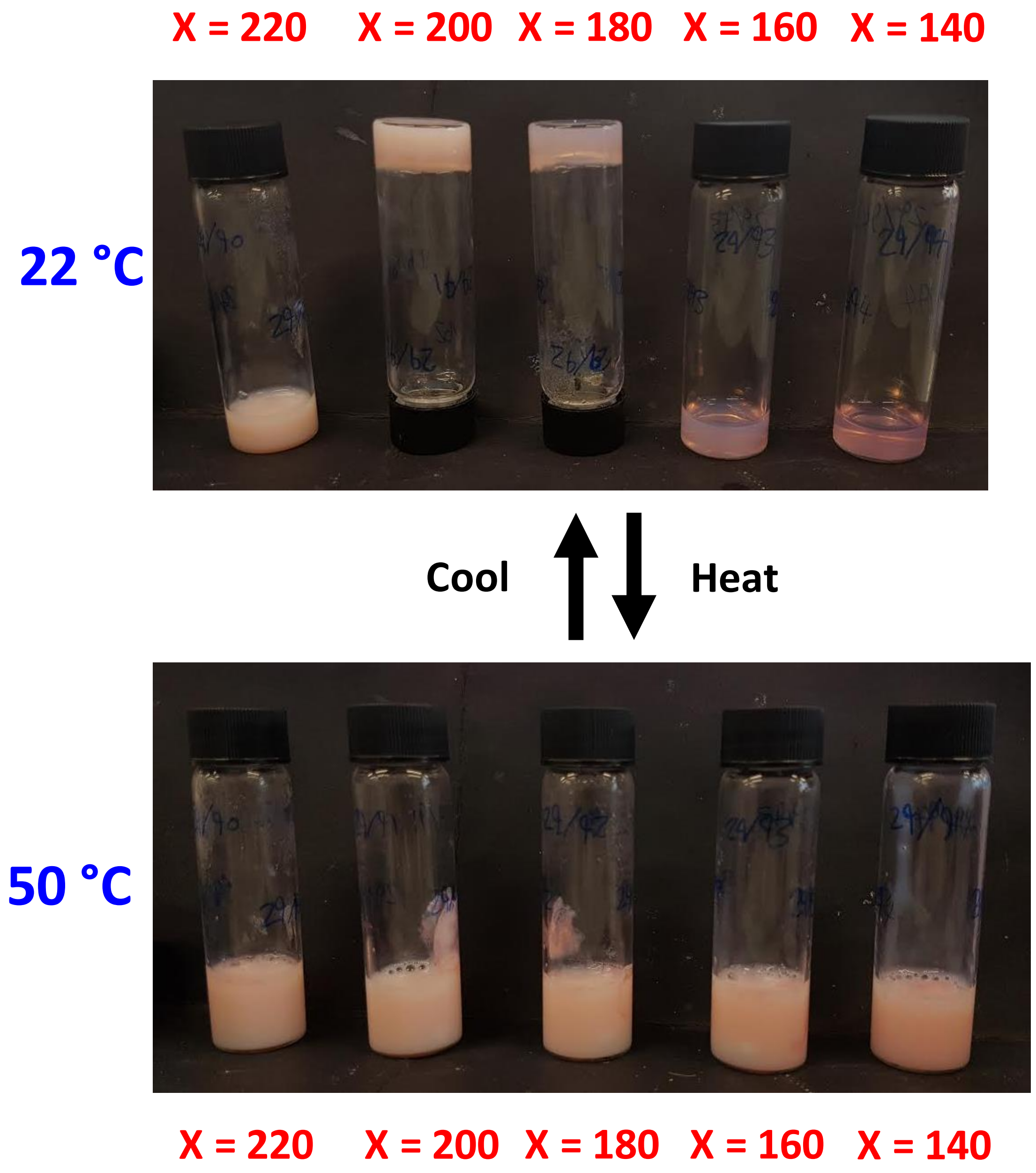

Figure S6. Digital photographs recorded for a series of 10% w/w aqueous dispersions of PHPMAC$_{41}$-PHPMA$_{x}$ (where x = 140, 160, 180, 200 or 220) diblock copolymer nano-objects at 22 °C and 50 °C (see **Figure S3** for the corresponding GPC data). Only PHPMAC$_{41}$-PHPMA$_{200}$ and PHPMAC$_{41}$-PHPMA$_{180}$ form free-standing gels at 22 °C, which indicate the presence of worms. PHPMAC$_{41}$-PHPMA$_{220}$ forms a turbid free-flowing dispersion of vesicles, while PHPMAC$_{41}$-PHPMA$_{160}$ is a mixture of spheres and worms and PHPMAC$_{41}$-PHPMA$_{140}$ forms weakly scattering spheres at 22 °C (these morphological assignments were confirmed by TEM studies – data not shown). However, all five copolymers form turbid, free-flowing vesicular dispersions at 50 °C. This confirms that the critical temperature for the formation of the worm gel phase is sensitive to the diblock copolymer composition, as expected.

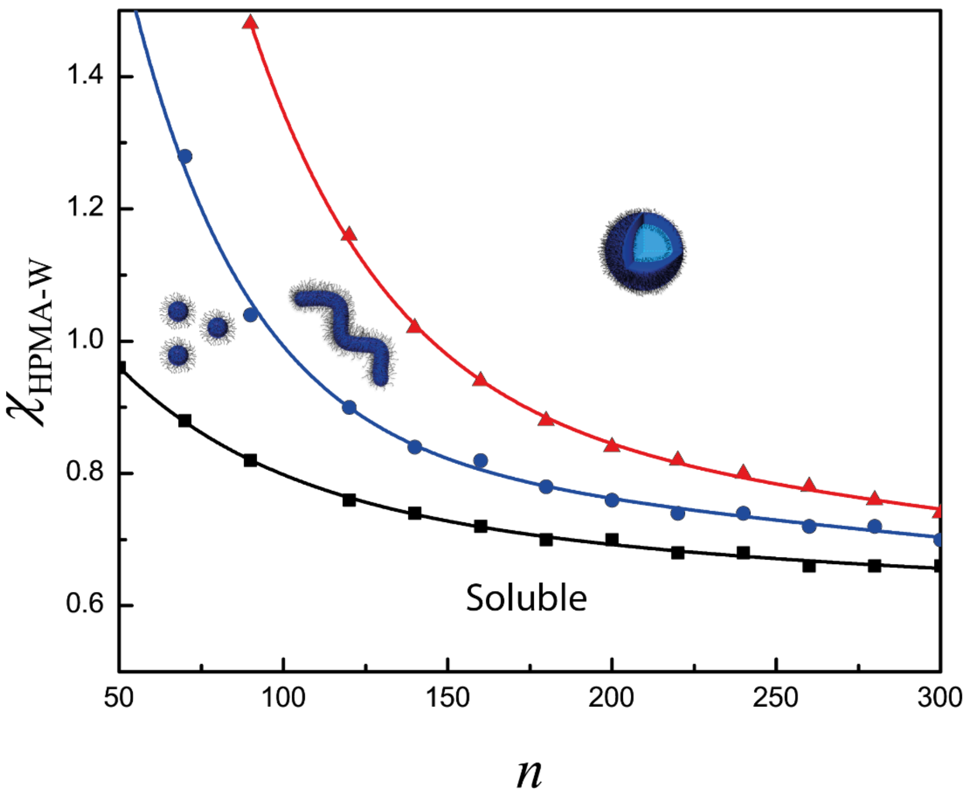

Figure S7. Theoretical phase diagram computed using self-consistent field (SCF) theory illustrating the preferred copolymer morphologies as a function of the PHPMA DP (*n*) and χ_{HPMA-W}.

Table S1. Summary of parameters obtained when fitting SAXS data to appropriate models for the coexistence of spherical micelles[8] and dissolved copolymer chains[9] at 4 °C, worm-like micelles[8] at 22 °C and vesicles[10] at 50 °C. R_g is the radius of gyration of the stabilizer block. D_sphere is the spherical nanoparticle diameter ($D_\mathrm{sphere} = 2R_\mathrm{s} + 4R_\mathrm{g}$, where R_s is the core radius). T_worm is the worm thickness ($T_\mathrm{worm} = 2R_\mathrm{w} + 4R_\mathrm{g}$, where R_w is the worm core cross-sectional radius). L_worm is the worm length. D_vesicle is the overall vesicle diameter ($D_\mathrm{vesicle} = R_\mathrm{m} + T_\mathrm{m} + 4R_\mathrm{g}$, where R_m is the radius from the centre of the vesicle to the centre of the membrane and T_m is the membrane thickness). x_sol is the volume fraction of solvent (water) within the structure-directing block. N_agg is the mean aggregation number, or number of copolymer chains per nanoparticle. $R_{\mathrm{g}_\mathrm{cop}}$ is the radius of gyration of dissolved copolymer chains.

**Temperature / °C**	R_g **/ nm**	D_sphere **/ nm**	T_worm **/ nm**	L_worm **/ nm**	D_vesicle **/ nm**	T_m **/ nm**	x_sol	N_agg	$R_{\mathrm{g}_\mathrm{cop}}$ **/ nm**
4	2.49	24.6	-	-	-	-	0.38	113	3.56
22	2.30	-	30.6	554	-	-	0.25	4194	-
50	2.21	-	-	-	99.6	12.9	0	6763	-

SAXS Models

In general, the X-ray intensity scattered by a dispersion of nano-objects [usually represented by the scattering cross-section per unit sample volume, $\frac{d\Sigma}{d\Omega}(q)$] can be expressed as:

$$\frac{d\Sigma}{d\Omega}(q) = NS(q)\int_0^\infty \dots \int_0^\infty F(q, r_1, \dots, r_k)^2 \Psi(r_1, \dots, r_k) dr_1 \dots dr_k \qquad S12$$

where $F(q, r_1, \dots, r_k)$ is their form factor, $r_1, \dots, r_k$ is a set of *k* parameters describing the structural morphology, $\Psi(r_1, \dots, r_k)$ is the distribution function, $S(q)$ is the structure factor and *N* is the nano-object number density per unit volume expressed as:

$$N = \frac{\varphi}{\int_0^\infty \dots \int_0^\infty V(r_1, \dots, r_k)\, \Psi(r_1, \dots, r_k) dr_1 \dots dr_k} \qquad S13$$

where $V(r_1, \dots, r_k)$ is volume of the nano-object and φ is their volume fraction in the dispersion. It is assumed that $S(q) = 1$ at sufficiently low copolymer concentrations (e.g. 1.0% w/w).

Gaussian chain model

Generally, the scattering cross-section per unit sample volume for an individual Gaussian polymer chain can be expressed as:

$$\frac{d\Sigma}{d\Omega}(q) = \varphi(\Delta\xi)^2 V_{\mathrm{mol}} F_{\mathrm{mol}}(q) \qquad S14$$

where V_{mol} is the total molecular volume and $\Delta\xi$ is the excess scattering length density of the copolymer [$\Delta\xi = \xi_{\mathrm{PHPMAC-PHPMA}} - \xi_{\mathrm{H_2O}} = 1.88 \times 10^{-10}$ cm^{-2}], where the scattering length density of the copolymer, $\xi_{\mathrm{PHPMAC-PHPMA}} = \frac{DP_{\mathrm{PHPMAC}}\xi_{\mathrm{PHPMAC}} + DP_{\mathrm{PHPMA}}\xi_{\mathrm{PHPMA}}}{DP_{\mathrm{total}}} = 11.3 \times 10^{-10}$ cm^{-2} and the scattering length density of water, $\xi_{\mathrm{H_2O}} = 9.42 \times 10^{-10}$ cm^{-2}. The generalized form factor for a Gaussian polymer chain is given by:[9]

$$F_{\mathrm{mol}}(q) = \left[\frac{1}{\upsilon U^{1/(2\upsilon)}}\gamma\left(\frac{1}{2\upsilon}, U\right) - \frac{1}{\upsilon U^{1/\upsilon}}\left(\frac{1}{\upsilon}, U\right)\right] \qquad S15$$

where the lower incomplete gamma function is $\gamma(s, x) = \int_0^x t^{s-1}\exp(-t)\,dt$ and U is the modified variable:

$$U = (2\upsilon + 1)(2\upsilon + 2)\frac{q^2 {R_{g_{cop}}}^2}{6} \qquad S16$$

Here, υ is the extended volume parameter (theta conditions were assumed and thus υ was fixed at 0.50) and $R_{g_{cop}}$ is the radius of gyration of the copolymer chain.

Spherical micelle model

The spherical micelle form factor for Equation S12 is given by:[8]

$$F_{s\_mic}(q) = {N_s}^2{\beta_s}^2{A_s}^2(q, R_s) + N_s{\beta_c}^2F_c(q, R_g) + N_s(N_s - 1){\beta_c}^2{A_c}^2(q) + 2{N_s}^2\beta_s\beta_cA_s(q, R_s)A_c(q) \qquad S17$$

where R_s is the radius of the spherical micelle core, R_g is the radius of gyration of the PHPMAC coronal block, the core block and the corona block X-ray scattering length contrast is given by $\beta_s = V_s(\xi_s - \xi_{sol})$ and $\beta_c = V_c(\xi_c - \xi_{sol})$, respectively. Here ξ_s, ξ_c and ξ_{sol} are the X-ray scattering length densities of the core block (ξ_{PHPMA} = 11.11 × 10^{10} cm^{-2}), the corona block (ξ_{PHPMAC} = 11.37 × 10^{10} cm^{-2}) and the solvent (ξ_{sol} = 9.42 × 10^{10} cm^{-2}), respectively. V_s and V_c are volumes of the core block (V_{PHPMA}) and the corona block ($V_{PHPMAC41}$), respectively. The volumes were obtained from $V = \frac{M_{n,pol}}{N_A\rho}$ using the solid-state densities of PHPMA (ρ_{PHPMA} = 1.21 g cm^{-3}) and PHPMAC (ρ_{PHPMAC} = 1.23 g cm^{-3}), where $M_{n,pol}$ corresponds to the number-average molecular weight of the block determined by ^{1}H NMR spectroscopy. The sphere form factor amplitude is used for the amplitude of the core self-term:

$$A_c(q, R_s) = \Phi(qR_s)\exp\left(-\frac{q^2\sigma^2}{2}\right) \qquad S18$$

where $\Phi(qR_s) = \frac{3[\sin(qR_s) - qR_s\cos(qR_s)]}{(qR_s)^3}$. A sigmoidal interface between the two blocks was assumed for the spherical micelle form factor Equation S18. This is described by the exponent term with a width σ accounting for a decaying scattering length density at the membrane surface. This σ value was fixed at 2.5 during fitting.

The form factor amplitude of the spherical micelle corona is:

$$A_{\mathrm{c}}(q)=\frac{\int_{R_{\mathrm{s}}}^{R_{\mathrm{s}}+2s}\mu_{\mathrm{c}}(r)\frac{\sin(qr)}{qr}r^{2}dr}{\int_{R_{\mathrm{s}}}^{R_{\mathrm{s}}+2s}\mu_{\mathrm{c}}(r)r^{2}dr}\exp\left(-\frac{q^{2}\sigma^{2}}{2}\right) \qquad S19$$

The radial profile, $\mu_c(r)$, can be expressed by a linear combination of two cubic b splines, with two fitting parameters *s* and *a* corresponding to the width of the profile and the weight coefficient, respectively. This information can be found elsewhere,[11] as can the approximate integrated form of Equation S19. The self-correlation term for the corona block is given by the Debye function:

$$F_{\mathrm{c}}(q,R_{\mathrm{g}})=\frac{2\left[\exp\left(-q^{2}R_{\mathrm{g}}{}^{2}\right)-1+q^{2}R_{\mathrm{g}}{}^{2}\right]}{q^{4}R_{\mathrm{g}}{}^{4}} \qquad S20$$

where R_g is the radius of gyration of the PHPMAC coronal block. The aggregation number of the spherical micelle is:

$$N_{\mathrm{s}}=(1-x_{\mathrm{sol}})\frac{\frac{4}{3}\pi R_{\mathrm{s}}{}^{3}}{V_{\mathrm{s}}} \qquad S21$$

where x_{sol} is the volume fraction of solvent in the PHPMA micelle core. A polydispersity for one parameter (R_s) is assumed for the micelle model which is described by a Gaussian distribution. Thus, the polydispersity function in Equation S12 can be represented as:

$$\Psi(r_{1})=\frac{1}{\sqrt{2\pi\sigma_{Rs}{}^{2}}}\exp\left(-\frac{(r_{1}-R_{\mathrm{s}})^{2}}{2\sigma_{Rs}{}^{2}}\right) \qquad S22$$

where σ_{Rs} is the standard deviation for R_s. In accordance with Equation S13 the number density per unit volume for the micelle model is expressed as:

$$N=\frac{\varphi}{\int_{0}^{\infty}V(r_{1})\Psi(r_{1})dr_{1}} \qquad S23$$

where φ is the total volume fraction of copolymer in the spherical micelles and $V(r_1)$ is the total *volume* of copolymer in a spherical micelle $[V(r_1)=(V_{\mathrm{s}}+V_{\mathrm{c}})N_{\mathrm{s}}(r_1)]$.

Worm-like micelles

The worm-like micelle form factor for Equation S12 is given by:[8]

$$F_{\mathrm{w\_mic}}(q) = {N_{\mathrm{w}}}^2{\beta_{\mathrm{s}}}^2F_{\mathrm{sw}}(q) + N_{\mathrm{w}}{\beta_{\mathrm{c}}}^2F_{\mathrm{c}}(q, R_{\mathrm{g}}) + N_{\mathrm{w}}(N_{\mathrm{w}} - 1){\beta_{\mathrm{c}}}^2S_{\mathrm{cc}}(q) + 2{N_{\mathrm{w}}}^2\beta_{\mathrm{s}}\beta_{\mathrm{c}}S_{\mathrm{sc}}(q) \quad \text{S24}$$

where β_{s} and β_{c} are as previously defined. The self-correlation term for the worm-like micelle core or radius R_{sw} is:

$$F_{\mathrm{sw}}(q) = F_{\mathrm{worm}}(q, L_{\mathrm{w}}, b_{\mathrm{w}}){A_{\mathrm{cs_{worm}}}}^2(q, R_{\mathrm{sw}}) \quad \text{S25}$$

which is a product of a core cross-section term:

$$F_{\mathrm{cs_{worm}}}(q, R_{\mathrm{g}}) = {A_{\mathrm{cs_{worm}}}}^2(q, R_{\mathrm{sw}}) = \left[2\frac{J_1(qR_{\mathrm{sw}})}{qR_{\mathrm{sw}}}\right]^2 \quad \text{S26}$$

where J_1 is the first-order Bessel function of the first kind, and a form factor $F_{\mathrm{worm}}(q, L_{\mathrm{w}}, b_{\mathrm{w}})$ for self-avoiding semi-flexible chains represents the worm-like micelle, where b_w is the worm Kuhn length and L_w is the mean worm contour length. A complete expression for the chain form factor can be found elsewhere.[12] The self- correlation term for the corona block is given by the Debye function shown in Equation S20. The interference cross-term between the worm micelle core and the corona chain is given by:

$$S_{\mathrm{sc}}(q) = \Psi^2(qR_{\mathrm{g}}){J_0}^2[q(R_{\mathrm{sw}} + R_{\mathrm{g}})]F_{\mathrm{worm}}(q, L_{\mathrm{w}}, b_{\mathrm{w}}) \quad \text{S27}$$

where $\Psi(qR_{\mathrm{g}}) = \frac{1-\exp(-q^2{R_{\mathrm{g}}}^2)}{(qR_{\mathrm{g}})^2}$ is the form factor amplitude of the corona chain, R_g is the radius of gyration of the PSMA corona block and J_0 is the zero-order Bessel function of the first kind. The interference term between the worm corona chains is:

$$S_{\mathrm{cc}}(q) = \Psi(qR_{\mathrm{g}})A_{\mathrm{cs\_worm}}J_0[q(R_{\mathrm{sw}} + R_{\mathrm{g}})]F_{\mathrm{worm}}(q, L_{\mathrm{w}}, b_{\mathrm{w}}) \quad \text{S28}$$

The mean aggregation number of the worm-like micelle is given by:

$$N_{\mathrm{w}} = (1 - x_{\mathrm{sol}})\frac{\pi {R_{\mathrm{sw}}}^2L_{\mathrm{w}}}{V_{\mathrm{s}}} \quad \text{S29}$$

where x_{sol} is the volume fraction of solvent within the worm-like micelle core. Possible semi-spherical caps at the ends of each worm are not considered in this form factor.

Vesicles

The vesicle form factor in Equation S12 is expressed as:[10]

$$F_{\mathrm{ves}}(q) = {N_{\mathrm{v}}}^2{\beta_{\mathrm{m}}}^2{A_{\mathrm{m}}}^2(q) + N_{\mathrm{v}}{\beta_{\mathrm{vc}}}^2F_{\mathrm{c}}(q, R_{\mathrm{g}}) + N_{\mathrm{v}}(N_{\mathrm{v}} - 1){\beta_{\mathrm{vc}}}^2{A_{\mathrm{vc}}}^2(q) + 2{N_{\mathrm{v}}}^2\beta_{\mathrm{m}}\beta_{\mathrm{vc}}A_{\mathrm{m}}(q)A_{\mathrm{vc}}(q) \qquad S30$$

where β_{s} and β_{c} are as previously defined. The amplitude of the membrane self-term is:

$$A_{\mathrm{m}}(q) = \frac{V_{\mathrm{out}}\varphi(qR_{\mathrm{out}}) - V_{\mathrm{in}}\varphi(qR_{\mathrm{in}})}{V_{\mathrm{out}} - V_{\mathrm{in}}}\exp\left(-\frac{q^2{\sigma_{\mathrm{in}}}^2}{2}\right) \qquad S31$$

where $R_{\mathrm{in}} = R_{\mathrm{m}} - \frac{1}{2}T_{\mathrm{m}}$ is the inner radius of the membrane, $R_{\mathrm{out}} = R_{\mathrm{m}} + \frac{1}{2}T_{\mathrm{m}}$ is the outer radius of the membrane (R_{m} is the radius from the centre of the vesicle to the centre of the membrane),, $V_{\mathrm{in}} = \frac{4}{3}\pi{R_{\mathrm{in}}}^3$, $V_{\mathrm{out}} = \frac{4}{3}\pi{R_{\mathrm{out}}}^3$. It should be noted that Equation S30 differs from the original work in which they were first described.[10] The exponent term in Equation S31 represents a sigmoidal interface between the blocks, with a width σ_{in} accounting for a decaying scattering length density at the membrane surface. The value of σ_{in} was fixed at 2.5. The mean vesicle aggregation number, N_{v}, is given by:

$$N_{\mathrm{v}} = (1 - x_{\mathrm{sol}})\frac{V_{\mathrm{out}} - V_{\mathrm{in}}}{V_{\mathrm{m}}} \qquad S32$$

where x_{sol} is the solvent (i.e. mineral oil) volume fraction within the vesicle membrane.

A simpler expression for the corona self-term of the vesicle model than for the spherical micelle corona self-term was used due to the fact that the contribution to the scattering intensity from the corona block in this case was much less than the contribution from the membrane block. Assuming that there is no penetration of the solvophilic coronal blocks into the solvophobic membrane, the amplitude of the vesicle corona self-term is expressed as:

$$A_{\mathrm{vc}}(q) = \Psi(qR_{\mathrm{g}})\frac{1}{2}\left[\frac{\sin[q(R_{\mathrm{out}} + R_{\mathrm{g}})]}{q(R_{\mathrm{out}} + R_{\mathrm{g}})} + \frac{\sin[q(R_{\mathrm{in}} - R_{\mathrm{g}})]}{q(R_{\mathrm{in}} - R_{\mathrm{g}})}\right] \qquad S33$$

where the term outside the square brackets is the factor amplitude of the corona block polymer chain such that:

$$\Psi(qR_g) = \frac{1 - \exp(-qR_g)}{(qR_g)^2} \qquad S34$$

It was assumed for the vesicle model that two parameters are polydisperse: the radius from the centre of the vesicle to the centre of the membrane (R_m) and the membrane thickness (T_m). They are considered to have a Gaussian distribution and, therefore, the polydispersity function in Equation S12 can be expressed as:

$$\Psi(r_1, r_2) = \frac{1}{\sqrt{2\pi\sigma_{Rm}^2}} \exp\left(-\frac{(r_1 - R_m)^2}{2\sigma_{Rm}^2}\right) \frac{1}{\sqrt{2\pi\sigma_{Tm}^2}} \exp\left(-\frac{(r_1 - T_m)^2}{2\sigma_{Tm}^2}\right) \qquad S35$$

where σ_{Rm} and σ_{Tm} are the standard deviations for R_m and T_m, respectively. Following Equation S13 the number density per unit volume for the vesicle model is expressed as:

$$N = \frac{\varphi}{\int_0^\infty \int_0^\infty V(r_1, r_2)\Psi(r_1, r_2)dr_1 dr_2} \qquad S36$$

where φ is the total *volume fraction* of copolymer in the vesicles and $V(r_1, r_2)$ is the total *volume* of copolymers in a vesicle $[V(r_1, r_2) = (V_m + V_{vc})N_v(r_1, r_2)]$.

References

[1] J. I. Jay, S. Shukair, K. Langheinrich, M. C. Hanson, G. C. Cianci, T. J. Johnson, M. R. Clark, T. J. Hope, P. F. Kiser, *Advanced Functional Materials* **2009**, *19*, 2969-2977.

[2] J. Ilavsky, P. R. Jemian, *J. Appl. Crystallogr.* **2009**, *42*, 347-353.

[3] J. Lyklema, in *Fundamentals of Interface and Colloid Science, Vol 5: Soft Colloids, Vol. 5*, **2005**, pp. 1-804.

[4] J. N. Israelachvili, D. J. Mitchell, B. W. Ninham, *Journal of the Chemical Society-Faraday Transactions* **1976**, *72*, 1525-1568.

[5] (a) S. Förster, M. Antonietti, *Advanced Materials* **1998**, *10*, 195-217; (b) A. Blanazs, S. P. Armes, A. J. Ryan, *Macromolecular Rapid Communications* **2009**, *30*, 267-277.

[6] (a) E. G. Kelley, T. P. Smart, A. J. Jackson, M. O. Sullivan, T. H. Epps, *Soft Matter* **2011**, *7*, 7094-7102; (b) T. Shikata, M. Okuzono, N. Sugimoto, *Macromolecules* **2013**, *46*, 1956-1961.

[7] E. E. Dormidontova, *Macromolecules* **2002**, *35*, 987-1001.

[8] J. S. Pedersen, *Journal of Applied Crystallography* **2000**, *33*, 637-640.

[9] B. Hammouda, *Probing Nanoscale Structures - The SANS Toolbox*, National Institute of Standards and Technology, **2008**.

[10] J. Bang, S. M. Jain, Z. B. Li, T. P. Lodge, J. S. Pedersen, E. Kesselman, Y. Talmon, *Macromolecules* **2006**, *39*, 1199-1208.

[11] (a) J. S. Pedersen, M. C. Gerstenberg, *Colloids and Surfaces A: Physicochemical and Engineering Aspects* **2003**, *213*, 175-187; (b) J. S. Pedersen, C. Svaneborg, K. Almdal, I. W. Hamley, R. N. Young, *Macromolecules* **2003**, *36*, 416-433.

[12] J. S. Pedersen, P. Schurtenberger, *Macromolecules* **1996**, *29*, 7602-7612.
